# “Cutting Down on Sugar” by Non-Dieting Young Women: An Impact on Diet Quality on Weekdays and the Weekend

**DOI:** 10.3390/nu10101463

**Published:** 2018-10-09

**Authors:** Magdalena Czlapka-Matyasik, Marta Lonnie, Lidia Wadolowska, Agnieszka Frelich

**Affiliations:** 1 Institute of Human Nutrition and Dietetics, Poznan University of Life Sciences, Wojska Polskiego 28, 60-637 Poznan, Poland; magdalena.matyasik@up.poznan.pl (M.C.-M.); frelich.a@interia.pl (A.F.); 2 Department of Human Nutrition, University of Warmia and Mazury in Olsztyn, Sloneczna 45f, 10-718 Olsztyn, Poland; lidia.wadolowska@uwm.edu.pl

**Keywords:** sugar restriction, diet quality, sucrose, sugar, weekend, weekdays, women

## Abstract

Restricted sugar intake is an important part of a healthy lifestyle and may contribute to the prevention of diet-related diseases. The aim of the study was to investigate whether declared sugar restriction is reflected in actual sucrose intake and diet quality of young non-dieting women, with differentiation between the weekend and weekdays. A convenient sample of 192 non-dieting women aged 20–30 years old was recruited. The sample was divided into two groups based on each woman’s declaration: “restricting sugar” (RS, *n* = 76) and “not restricting sugar” (nRS, *n* = 116). Comparisons between groups were made separately for seven days of the week, five weekdays and two weekend days without and with an adjustment for 2000 kcal of energy. Relative differences (RD, in %) in mean diet nutritional values between groups were calculated, where RD (%) = (RS − nRS) × 100/nRS, and were verified with a two-tailed *t*-test. In the seven-day diet of the RS group, in comparison to the nRS group, a lower daily intake of sucrose (by 22%) and MUFA (by 8%) and a higher content of most nutrients (by 7–38%) was found. No significant differences were observed in energy intake between RS and nRS women over the course of seven days, including weekdays and the weekend. Daily intake for the weekend, in comparison to weekdays, in RC and nRC women was higher with respect to energy (by 530 and 512 kcal, i.e., 37% and 34%, respectively), sucrose (44% and 23%, respectively), and most nutrients (by 17–98% and 16–42%, respectively). However, after the adjustment for 2000 kcal of energy, the daily intake of most nutrients in the weekend was lower (by 6–30% and 3–27%, in RS and nRS groups, respectively), than during weekdays. The intention to restrict sucrose has improved diet quality by decreasing sucrose intake and increasing the content of most nutrients, but had no effect on energy intake throughout the week. The quality of the women’s diet during the weekend was compromised, regardless of restricting or not restricting sugar. Encouragement to restrict sugar intake throughout the week and control the food intake during the weekend may be an effective strategy for young women to maintain a healthy diet.

## 1. Introduction

Sugar consumption is increasing globally, including in Poland [1,2]. Over the past decade, sugar intake in the Polish population increased by 10%, accounting for 42.3 kg per capita/per annum in 2016 (approx. 115 g per person/day) [2]. This indirect method of sugar intake assessment is based on the reported annual food availability for consumption and does not provide data regarding the actual intake in age- and sex-specific groups. However, these rough estimates still suggest that sugar intake in Poland highly exceeds WHO’s recommended level of added sugar intake, which is less than 50 g per day (<10% of total energy intake) [3]. Interestingly, the structure of sugar consumption in Poland has been evolving over recent years. While consumers buy less table sugar, the consumption of sugar added as an ingredient to processed foods has been increasing [2]. This worrying trend may indicate gaps in consumers’ nutritional knowledge or confusion around the actual content of sugar found in commonly consumed foods [4].

Excessive sugar intake can directly and indirectly contribute to cardiovascular diseases and type 2 diabetes [5]. The direct way would be through the metabolic pathways of sugar in human body. For example, sugar consumed in a liquid form (sweetened beverages) increases the rate of hepatic extraction of fructose, accelerating the lipogenesis processes [6]. Moreover, a high glycemic load of foods high in sugar trigger a higher post-prandial insulin response, which may result in hyperinsulinemia or insulin resistance [7]. Indirectly, high sugar intake contributes to a positive energy balance leading to obesity [8], which is common mediator of type 2 diabetes, metabolic syndrome, and cardiovascular diseases [9,10]. A meta-analysis by Te Morenga et al. [11] revealed that sugar intake is an independent determinant of body weight. In this study, no difference in weight gain was observed when sugar was isoenergetically exchanged with other carbohydrates or macronutrients, leading to a conclusion that change in body weight is most likely attributed to the calorific load of sugar, and not necessarily its biochemistry.

Lastly, the reduction of sugar intake in a young women’s diet is of particular importance to the health of their children, starting from the early stages of life. The emerging evidence links maternal free-sugar intake in pregnancy with an increased risk of atopy and atopic asthma [12] and poorer cognition [13] in the offspring. Concerning is also a high sugar intake by lactating mothers, which started a debate about “secondhand sugars”. A recent study has shown, excessive fructose in breast milk is positively associated with all components of body composition in infants [14].

Therefore, health messages encouraging people to “cut down on sugar” has been actively promoted lately through media and public health interventions [15,16]. One of the strategies to comply with this dietary recommendation is self-regulation. As reported in our previous work, the most commonly reported dietary restriction in young Polish women regarded sugar and sweets intake (23.7% of a representative national sample of female aged 13–21 years) [17]. Since self-regulation of sugar intake was associated with higher adherence to a pro-healthy dietary pattern, this type of dietary restraint may be perceived as a desired health behavior. However, the above-mentioned study used a semi-quantitative method for dietary intake assessment, and it remains unclear whether declared sugar restriction is associated with energy intake and a nutritional profile. To provide greater veracity, a food record was used in the current study to assess the intake of energy and a wide selection of macro- and micronutrients. 

Another question, which to our knowledge has not been answered to date, is whether the declared sugar restriction is effective throughout the week. It has been previously reported that during the weekend, energy and sugar intake is higher and diet quality lower, in a population of healthy adults [18,19]. However, it has not yet been investigated whether this phenomenon is observed in young, non-dieting women who declare restricting sugar intake.

### Study Aims

This study aimed to investigate whether young, non-dieting women who declared restricting sugar intake had lower sucrose intake and a better quality diet than women who do not restrict sugar and whether this restriction is effective throughout the week.

## 2. Methods

The study was approved by the Bioethics Committee of the Faculty of Medical Sciences, University of Warmia and Mazury in Olsztyn on 17 June 2010, Resolution No. 20/2010. A written agreement to participate in the study was required. All data was collected by well-trained researchers during one-to-one appointments.

### 2.1. Study Design

**Figure 1:**
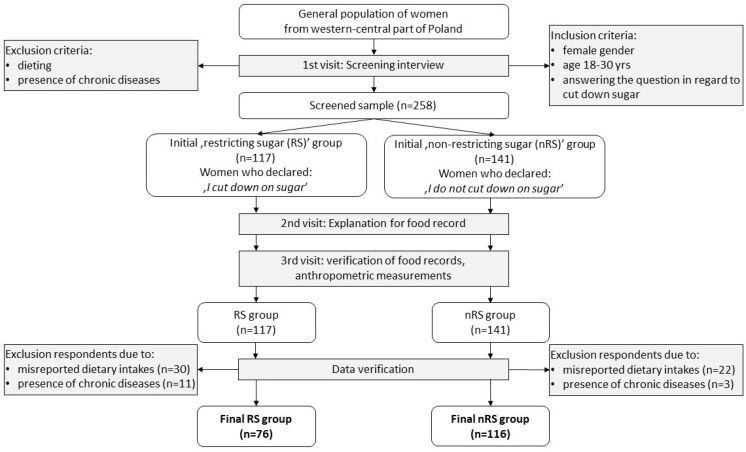
Study design and data collection.

The data for this cross-sectional study were collected from December 2013 to March 2014. During the first contact (first visit), subjects were screened with regard to inclusion/exclusion criteria. Each subject who met the criteria and agreed to take part in the study by providing informed consent was invited to meet the researcher on two further, consecutive occasions. During the second visit, templates of food records were distributed to respondents and a detailed explanation from trained researchers regarding the accurate completion was provided. During the third visit, records in food diaries were verified and anthropometric measurements were taken (see Figure 1 in Section 2.2).

### 2.2. Sample Recriutment

The participants were a convenient sample of female volunteers recruited through advertising at the university, local work places, and public spaces. The recruitment was carried out in the rural and urban area of western-central Poland. The inclusion criteria were: age (18–30 years), female gender, and answering the question: “do you cut down on sugar in your habitual diet?” (yes or no). It was clearly explained to respondents that reducing sugar intake is thought to be a part of habitual diet only and not through dieting. The exclusion criteria were: chronic diseases affecting dietary habits or dieting due to obesity, diabetes, celiac disease, or other diseases. 

Initially, 258 participants were recruited. Based on each woman’s declaration, participants were divided into two groups: “restricting sugar” (RS, *n* = 117) and “not-restricting sugar” (nRS, *n* = 141). During dietary data verification, 52 participants (RS/nRS 30/22) were excluded due to misreported dietary intakes, e.g., unrecorded meals or omitted days in food records or an incomplete record of food amount; significantly more respondents were excluded from RS than nRS group (*p* = 0.0461). To verify food records, no cut-offs with regard to energy intake were applied. A further 14 participants (11/3) were excluded due to a presence of chronic diseases such as diabetes, hyperthyroidism, stomach ulcer, celiac disease, bulimia, and endocrinological disorders. In total 66 participants (41/25) were excluded from the screened sample. The final study sample consisted of 192 non-dieting women (76/116) aged 20–30 years (Figure 1).

Sample size was calculated using the expected difference in daily sucrose intake between two groups of women, restricting and not restricting sugar intake. With a 5% significance level and 80% power, the minimum sample size required was approximately 118 respondents per group (assuming a 1:1 ratio for two groups) to detect a 20% difference in sucrose intake between two groups, including recoding error or missing data of 20%. An adequacy of sample size was checked for the data under study, and the post-hoc statistical power was calculated. When compared, the mean difference in daily sucrose intake between women restricting and not restricting sugar intake (76/116), assuming 5% significance level, the statistical power was 73.5%. Thus, we have found that the sample size was sufficient.

### 2.3. Dietary Data Collection

An estimated food record method was applied, covering seven consecutive days, five weekdays (Monday–Friday) and two weekend days (Saturday–Sunday) [20]. Respondents recorded the intake of all foods and beverages in paper food dairies continuously throughout the day. During the second visit, respondents were instructed to write down the type and brand name of the product, along with the weight (displayed on the product label) and time consumed. Alternatively, if the product was not entirely consumed or weight was unknown, the respondents were asked to write down portion size in household measures (e.g., small cup, little bowl, large plate). If the food was home-made, respondents were asked to record type, brand name, and weight of all ingredients along with a description of food preparation, cooking method (e.g., cooking, frying, grilling), and cooking time. If eating out, respondents were asked to record the type of the food, portion size, and name of the restaurant (if part of a chain). 

To resolve any doubts (e.g., regarding imprecise or missing inputs), all food records and completed questionnaires were verified by researchers during interviews with respondents (third visit) [20]. The amount of food was determined with the use of an “album of photographs of food products and dishes” [21] and expressed in grams. The mean daily energy and selected nutrients intake was calculated for each respondent using “Energia v.4.1” software (http://energia.waw.pl/) with an implemented food database. The food database was composed of 1178 products: 962 from the official database of foods commonly consumed in Poland [22] and 216 manual inputs (from the USDA database, [23]) of ethnic foods or products new on the market, which could not be found in the Polish “food composition tables” [22]. Plate waste was estimated using software built-in options and accounted for 10% of energy and all the macro- and micronutrients (10%), with the exception of vitamin C (55%), folic acid (40%), vitamin A (25%), B1 (20%), B2 (15%), and niacin (15%). The use of nutrient supplements was not taken into consideration.

### 2.4. Other Data Collection

Socioeconomic variables were collected using standard questions. A closed-question questionnaire was used. The questions included: place of residence (village, town, city), level of education (secondary, higher), and current occupation (student, office worker, physical worker, services worker). 

The measurements of body weight (kg) and height (cm) were taken between 8 and 12 a.m. during one-to-one appointments by trained staff, according to the International Standards for Anthropometric Assessment guidelines by International Society for the Advancement of Kinanthropometry (ISAK) [24]. Professional equipment was used. The measurement of body weight was taken in light indoor clothes without shoes and recorded with a precision of 0.1 kg. The measurement of height was taken with the head in horizontal Frankfort plane and recorded with a precision of 0.1 cm. Body mass index (BMI, kg/m^2^) was calculated [24]. The BMI was applied to assess the prevalence of underweight (<18.5 kg/m^2^), normal weight (18.5 to 24.9 kg/m^2^), overweight (25 to 29.9 kg/m^2^), and obesity (≥30 kg/m^2^) using WHO standards [25]. The body fat percentage (BF, %) was measured using a near-infrared spectrophotometry (NIR) method with Futrex 6100A/ZL (Futrex, Inc., Hagerstown, MD, USA). The measurement was made halfway between the antecubital fossa and the acromion, identified with a biceps locator.

### 2.5. Statistical Analysis

Continuous data are presented as means and 95% confidence intervals (95%CI), and categorical variables as sample percentages (%). The differences between groups were verified using a two-tailed *t*-test (continuous data) or *χ*^2^ test (categorical data) [26]. Variables’ normality was checked using a Kolmogorov–Smirnov test. All data were logarithmic transformed before analysis. All comparisons between groups were made separately for seven days of the week, five weekdays (“5 weekdays”) and two weekend days (“2 weekend days”) without and with the adjustment for 2000 kcal of energy. Relative differences (RD, in %) in mean diet nutritional value between groups: RS and nRS (Formula (1)) and “2 weekend days” and “5 weekdays” (Formula (2)) were calculated.

RD (%) = (RS − nRS) × 100/nRS(1)

RD (%) = (“2 weekend days” − “5 weekdays”) × 100/”5 weekdays”(2)

Notes: RS—"restricting sugar” (women who declared “*I cut down on sugar”)*, nRS—"not restricting sugar” (women who declared “*I don’t cut down on sugar*”), “2 weekend days”—mean daily intake calculated for two weekend days, “5 weekdays”—mean daily intake calculated for five weekdays. A *p*-value < 0.05 was considered significant. The statistical analysis was carried out using STATISTICA software (version 10.0 PL; StatSoft Inc., Tulsa, OK, USA; StatSoft Polska, Kraków, Poland).

## 3. Results

### 3.1. Sample Characteristics

The mean age of the study participants was 24.6 years old. The majority of women had a regular weight (71%), 22% were overweight or obese, and 7% were underweight. Most women lived in urbanized areas (92%), declared a secondary level of education (60%), and worked in the office environment (33%). No differences between RS and nRS groups were found regarding age, BMI, body fat, and socioeconomic variables (Table 1).

### 3.2. Differences between the Groups (Restricting vs. Not Restricting Sugar)

#### 3.2.1. Energy and Sucrose

Mean values of the daily dietary intake are presented in Supplementary Material (Table S1). No significant differences in energy intake between RS and nRS women were revealed (1570 and 1621 kcal/day, respectively) (Table S1). In the RS group, 66% of women had a sucrose intake of <10% of their daily energy. In the nRS group, a sucrose intake of <10% was observed in 34% of women (Table 2). 

Sucrose intake was significantly lower in the RS group (in crude and adjusted models respectively: by 22% and 20% over the course of seven days, 25% and 23% during weekdays and 12% and 11% at the weekend) (Table 3). 

#### 3.2.2. Diet Quality

The crude model revealed that diets of RS women over the course of seven days, five week days, and two weekend days (when compared to the nRS women) was significantly lower in MUFA (by 8%, 8%, and ns, respectively) and higher in β-carotene (38%, 41%, and 19%), fiber (30%, 29%, and 28%), calcium (26%, 28%, and 22%), magnesium (18%, 18%, and 16%), vitamin B2 (16%, 13%, and 19%), phosphorus (17%, ns, and 16%), vitamin C (17%, ns, and ns), folic acid (15%, 13%, and 16%), copper (14%, 15%, and ns), B12 (10%, ns, and 42%), zinc (10%, 10%, and ns), iron (8%, 8%, and ns), potassium (7%, 8%, and ns), protein (7%, ns, and ns). After the adjustment for 2000 kcal of energy, the diet of RS women was still higher in most nutrients than nRS women, although fewer differences were statistically confirmed (Table 3). 

### 3.3. Differences between Weekdays and Weekend (“2 Weekend Days” vs. “5 Weekdays”)

#### 3.3.1. Energy and Sucrose

Mean values of daily dietary intake are presented in the Supplementary Material (Table S1). Daily energy intake was significantly higher at the weekend than during the weekdays in both groups (by 530 and 512 kcal, i.e., 37% and 34%, RS and nRS, respectively) (Table S1, Table 4). In the crude model, sucrose daily intake at the weekend was higher by 44% and 23% in RS and nRS women, respectively, than during the weekdays. After adjustment for 2000 kcal, the difference in sucrose daily intake between weekend and weekdays in the RS women was no longer significant, and in nRS women it was lower by 8% (Table 4).

#### 3.3.2. Diet Quality

In the crude model, daily dietary intake of most nutrients was higher at the weekend than during the weekdays in both RS and nRS groups. At the weekend, in comparison to weekdays, higher daily intake of MUFA (by 43 and 41%, in RS and nRS, respectively), PUFA (39 and 42%), fat (by 39 and 35%), SFA (by 38 and 30%), phosphorus (by 33 and 17%), vitamin B1 (by 32 and 31%), carbohydrates (by 32 and 30%), vitamin E (by 31 and 39%), calcium (by 26 and 32%), cholesterol (by 26 and 16%), zinc (by 25% in both groups), sodium (by 25 and 22%), niacin (by 23 and 27%), magnesium (by 23 and 24%), iron (by 23% in both groups), vitamin B2 (by 23 and 17%), folic acid (by 22 and 19%), vitamin B6 (by 21 and 25%), copper (by 20 and 24%), fibre (by 20 and 22%), potassium (by 17 and 24%) was revealed. Also, in RS women, daily intake of retinol (by 98%) and vitamin D (by 34%) was higher at the weekend than weekdays (Table 4).

After the adjustment for 2000 kcal, the daily intake of most nutrients was lower at the weekend than during the weekdays in both groups of women. In the RS group, the decrease ranged from 6 to 30% and regarded the following nutrients (in the descending order): β-carotene, potassium, vitamin C, copper, fiber, magnesium, iron, vitamin B1, vitamin B2, niacin, zinc, sodium, calcium, and protein. In the nRS group the decrease ranged from 3 to 27% and regarded (in the descending order): retinol, vitamin B12, vitamin D, phosphorus, vitamin C, vitamin B2, cholesterol, folic acid, fiber, sodium, iron, potassium, magnesium, copper, vitamin B6, zinc, niacin, protein, and carbohydrates (Table 4).

## 4. Discussion

Our study revealed that young, non-dieting women who declared restricting sugar intake consumed less sucrose and more nutrients over the course of seven days than women who did not intend to self-regulate sugar intake. No differences were observed in energy intake between the groups. At the weekend, women from both groups consumed significantly more energy, sucrose, and other nutrients than during the weekdays. However, after the adjustment for 2000 kcal, the intake of most nutrient in both groups was lower than during weekdays, indicating that the weekend diet was abundant in calories and of low quality.

Over the course of seven days, women who declared cutting down on sugar consumed less sucrose (by 20 and 22%, in the crude and adjusted models, respectively) than women who did not attempt to regulate sugar intake, although no difference in energy intake between the groups was observed. Interestingly, 1/3 of women who declared restricting sugar had sucrose intake above the 10% of daily energy, suggesting that the perception of their own dietary behavior was not coherent with actions in this subset. High sucrose intake in women who restricted sugar intake from our study may have been a result of either low levels of the adherence to the intention, or was caused by low awareness about “hidden” sugars in products not typically associated with high sugar content, such as low-fat dairy, salad dressings, sauces, instant soups, ready meals, etc. It has been reported, that intention (directly) and personally-endorse motives (indirectly) predict sugar intake [28]. Perhaps intention not supported by nutritional knowledge was not sufficient to maintain sugar intake within the recommendations. Recent studies report that consumers find the latest dietary recommendations confusing and are unable to identify the differences between total, added, and free sugars [29,30]. Current labelling systems may, therefore, be misleading, e.g., a “no added sugars” statement on fruit juice or smoothies may falsely suggest a low total sugar content of the product. Therefore, self-regulation reinforced with nutrition education (e.g., label reading) could be an effective strategy to lower sugar intake [31,32]. 

No differences in energy intake between women restricting and not restricting sugar observed in our study can be explained by the “seesaw” effect. The term was originally used by Drewnowski et al. [33] to describe the phenomenon of compensating lower sucrose intake with energy from fats. In our study, we did not observe a higher intake of fat in the “restricting sugar” group, in comparison to the “not restricting sugar” group. However, the intake of protein was 11% higher (seven days, model adjusted for 2000 kcal) in the “restricting sugar” group, suggesting that protein could have been the energy-compensating macronutrient in our sample. This finding corresponds with the emerging trend that highlights the crucial role of protein in muscle health and appetite control [34]. Also, along with sugar restriction, women may have been cautious regarding foods containing fats, despite such declarations not being sought from study participants.

Over the course of seven days, women who restricted sugar intake had a better diet quality than those with no restriction. Considering that the sample included only non-dieting participants, sugar restriction was most likely health-oriented. Our findings are consistent with previous reports. People with strong healthy eating motivation display healthier lifestyles than those with weak motivations [35]. An inverse association was found in adults who do not seem to restrict their dietary intakes. A review by Louie and Tapsell [36] revealed that in 21 out of 22 studies, higher intake of added sugars was associated with a low-quality diet, and in 21 out of 30 studies, intake of added sugars was associated with a lower micronutrient intake. It is possible that by applying just one change in a habitual diet (i.e., sugar intake), the awareness was also shifted toward other food choices, improving the overall diet quality. It has been previously shown that sugar and sweets restrictions in young women are associated with more frequent fruit and vegetable intake [17]. In our study, women who restricted sugar had a 30% higher fiber intake than the non-restricting group, which may reflect a similar substitution in our sample. Also, one way to overcome sugar cravings may be to snack on fruit, either fresh or dried (e.g., raisins, dates, and prunes). Despite high fructose content, this replacement may still be perceived as beneficial, being a good source of micronutrients, antioxidants and fiber.

The diet quality of women from both groups was compromised during the weekend and contained significantly more energy (by over 500 kcal/day). The over-indulgence during the weekend is a finding well-established in the literature. A study by An [18] found that U.S. adults were eating less healthily at the weekend, with diet quality being the poorest on Saturdays. The energy intake of women from that study increased by 155 kcal on Saturday and 76 kcal on Sunday when compared to weekdays. The weekend diet in this sample was also characterized by lower fiber and fruit and vegetable intake, and a higher intake of fat, saturated fat, cholesterol, and sodium. No differences were observed in sugar consumption [18]. A similar observation was made by Racette et al. [19], but in a group of calorie-restricted adults aged 50–60 years old, in which a higher intake of energy and fat, but not carbohydrates and protein, was recorded on Saturdays. Correspondingly, women from our study, after the adjustment for 2000 kcal, consumed less protein (both groups), sucrose, and carbohydrates (only “not restricting sugar” group) at the weekend; however, no increase in the intake of fat was observed in the adjusted model. These findings imply that individuals who try to control their dietary intake and those who do not overindulge at the weekend. However, sugar and/or carbohydrates are not the main energy contributor during these days. 

In our study, an increased intake of most nutrients was reported during the weekend in both groups. However, the adjustment for 2000 kcal of energy intake proved that it was most likely a result of overeating since the decrease in relative differences was observed in the intake of majority of nutrients (by 3 to 30% and 3 to 27%, in “restricting sugar” and “not restricting sugar” groups, respectively). In the “restricting sugar” group, the greatest decrease observed regarding β-carotene intake. Considering that fruit and vegetables are primary dietary sources of this pro-vitamin, it can be assumed that foods from this group were less frequently consumed at the weekend. In the “not restricting sugar” group, after the adjustment for 2000 kcal, the greatest decrease was observed in terms of retinol, vitamin A, and vitamin B12 intake, which suggested a lower intake of animal-originated foods at the weekend. These foods could have been substituted with alcohol. Due to software limitations, the percentage of energy from alcohol intake was not possible to extract from our database. However, a parallel analysis of the food frequency consumption (data not shown) revealed that alcohol intake in our study sample corresponded with a frequency of “few times per month” to “once a week”. Hence, the increased intake of alcohol on one of the weekend days could not be overruled. Another explanation for the increased calorie load could be attributed to a higher intake of processed plant-foods (e.g., dumplings, crisps, chips, and pretzels) consumed while watching television or socializing at the weekend.

Although the quality of diet was lower at the weekend in both groups of women, it has to be stressed that the “restricting sugar” group had still a better nutrient profile than the “not restricting sugar” group. After the adjustment for 2000 kcal, during the weekend, women who restricted sugar had higher intakes of fiber (by 30%), protein (by 9%), vitamins (by up to 52%), and minerals (by up to 27%) than the “not restricting sugar” group. This indicates that the weekend diet of women who declared cutting down on sugar was slightly more favorable, despite the overindulgence.

## 5. Limitations

Although the research has reached its aims, we are aware that it may have some limitations. First, as the focus of the study was on sucrose intake, other potential restrictions have not been investigated. It cannot be ruled out that other modifications were present in women’s diet, e.g., restricting salt or fat and/or cholesterol intake [17,37]. The picture is thus still incomplete. However, this is the first study that looked into the associations between the intended reduction in sugar intake and diet quality in young women over the course of a week. We hope that our research will serve as a base for future studies to reveal which restrictions cluster in non-dieting women and what are their cumulative vs. singular effects on a diet’s quality during the week days and weekend. 

Second, since data were collected only during winter months, our study has not considered the seasonality of food consumption. Although seasonal variations can be significant in some specific groups [38,39], it has been shown, that this effect had a decreasing magnitude over the last 20 years, most likely due to socioeconomical changes, easier food access, and improved insulation of homes and workplaces [40]. 

Third, we are aware of limitations related to food recording as a method chosen to assess dietary intake, in particular the risk of underreporting. In our study, the mean reported energy intake was 1601 kcal/day. The daily resting energy expenditure of women in our study sample calculated with the Mifflin–St Jeor equation [41] was 1381 kcal. Considering low physical activity of young Polish women reported by WHO [42] and average (non-athletic) body fat percentage [43] of women in our study, we can assume that the level of physical activity was indeed low, resulting in the total energy expenditure to be around 1930 kcal/day. This estimation suggests the underreporting of approximately 330 kcal/day (17%), which is in line with previous studies. Results of the meta-analysis by Polusna et al. [44] revealed that the underreporting in estimated food records ranges from 7–20%, and is higher in women. The reported energy intake did not differ significantly between the groups (RS vs. nRS), which leads to the assumption that level of underreporting was similar in both groups. Therefore, the comparisons made between the groups were unlikely to be biased by this factor.

Fourth, although the minimum sample size criteria were met in this study, our investigations have only been on a small scale. Perhaps a larger sample size would reveal more significant differences between the groups, allowing stronger conclusions. Concerning might also be a significantly higher number of women who were excluded from the RS group, due to misreporting. It could be that women in the RS group were more prone to omit inputs in the food dairies more frequently than women from the nRS, due to concerns of how they will be perceived if they input foods that were incoherent with the sugar intake declaration. However, the exclusion of these women was necessary to minimize the risk of bias in this group. Finally, our results are limited to young women and may be not applicable to men.

## 6. Conclusions

The intention to cut down on sugar has improved the quality of women’s diets by decreasing sucrose intake and increasing the nutrients content, but no impact on energy intake was shown. Irrespective of restricting or not restricting sugar intake, weekend diets were characterized by high energy (by over 500 kcal/day), sucrose, and nutrient content. However, when adjusted for 2000 kcal, diet quality in both groups was lower than during weekdays. To improve overall diet quality, two recommendations can be made. First, to encourage young women to restrict sugar intake in their habitual diet, and second, to monitor diet quality during the weekend.

## Figures and Tables

**Table 1:** Sample characteristics (*n* = 192).

Category	Total (*n* = 192)	RS (*n* = 76)	nRS (*n* = 116)	*p*
Age ^#^ (years)	24.6 (24.3, 25.0)	24.4 (23.8, 24.9)	24.8 (24.3, 25.3)	ns
Weight	62.3 (61.0, 63.5)	62.5 (60.6, 64.4)	62.2 (60.5, 63.9)	ns
Height	166.7 (165.9, 169.4)	166.2 (165.0, 167.4)	166.9 (166.0, 167.8)	ns
BMI ^#^ (kg/m^2^)	22.5 (22.0, 22.9)	22.6 (22.0, 23.3)	22.3 (21.7, 22.9)	ns
BMI ^§^ (%)				ns
Underweight	7	3	10	
Regular weight	71	79	66	
Overweight	20	16	23	
Obesity	2	2	2	
Body fat (%)	24.7 (24.1, 25.3)	25.0 (23.7, 25.3)	24.5 (23.7, 25.3)	ns
Residence (%)				ns
Village	8	5	10	
Town	59	67	53	
City	33	26	37	
Education (%)				ns
Secondary	60	53	64	
Higher	40	47	36	
Profession (%)				ns
Student	25	26	24	
Office worker	33	38	30	
Physical worker	20	18	21	
Services worker	22	18	25	

Restricting sugar (RS), not restricting sugar (nRS); ^#^ Data are expressed as percentage or mean and the 95% confidence interval is given in brackets; ^§^ Underweight (<18.5 kg/m^2^), normal weight (18.5 to 24.9 kg/m^2^), overweight (25 to 29.9 kg/m^2^), and obesity (≥30 kg/m^2^) [25]; *p*-value, *t*-Student test, or *χ*^2^ test significance level is given after logarithmic data transformation; ns–insignificant differences; *p*-value < 0.05 was considered statistically significant.

**Table 2:** Comparison of sample distribution (in %) by low and high sucrose intake.

		Total	Low Sucrose Intake <10% ^§^ of Daily Energy	High Sucrose Intake ≥10% of Daily Energy	*p*
		*n* = 192	*n* = 89	*n* = 103	
RS	*n* = 76	100	66	34	****
nRS	*n* = 116	100	34	66	
Total	*n* = 192	100	46	54	

RS (“restricting sugar”)—women who declared “*I do cut down on sugar*”, nRS (“not restricting sugar”)—women who declared “*I don’t cut down on sugar*”; ^§^ based on Polish and WHO recommendation, which are related to added sugars or free sugars [3,27]. The content of free sugars other than sucrose was not considered. **** *p* < 0.0001.

**Table 3:** Relative differences (RD, in %) in mean daily dietary intakes between women “restricting sugar” (RS) and “not restricting sugar” (nRS) over seven days, including five weekdays and two weekend days, with crude and adjusted models.

Nutrient	Crude (RS vs. nRS)						Adjusted for 2000 kcal (RS vs. nRS)					
	7 Days		5 Week Days		2 Weekend Days		7 Days		5 Week Days		2 Weekend Days	
	RD	*p*	RD	*p*	RD	*p*	RD	*p*	RD	*p*	RD	*p*
Energy	−3	ns	−4	ns	−2	ns	-	-	-	-	-	-
Sucrose	−22	***	−25	***	−12	**	−20	***	−23	***	−11	*
Protein	7	*	6	ns	6	ns	11	***	12	***	9	**
Fat	−5	ns	−6	ns	−3	ns	−2	ns	−2	ns	−1	ns
SFA	−6	ns	−8	*	−2	ns	−3	ns	−4	*	0	ns
MUFA	−8	*	−8	*	−7	ns	−5	*	−4	ns	−5	ns
PUFA	0	ns	1	ns	−1	ns	4	ns	5	ns	1	ns
Cholesterol	0	ns	−3	ns	5	ns	3	ns	2	ns	6	ns
Carbohydrates	−3	ns	−4	ns	−2	ns	0	ns	0	ns	0	ns
Fibre	30	***	29	***	28	***	35	***	35	***	30	**
Sodium	−3	ns	−3	ns	−1	ns	2	ns	3	ns	2	ns
Potassium	7	*	8	*	2	ns	12	***	14	***	5	ns
Calcium	26	***	28	***	22	**	32	***	34	***	27	***
Iron	8	*	8	*	8	ns	13	***	13	***	10	*
Zinc	10	**	10	*	9	ns	15	***	15	***	11	**
Phosphorus	17	***	2	ns	16	**	22	***	8	ns	19	***
Magnesium	18	***	18	***	16	**	22	***	23	***	18	***
Copper	14	**	15	**	11	ns	18	***	20	***	13	**
Vitamin B1	0	ns	−1	ns	0	ns	4	ns	5	ns	2	ns
Vitamin B2	16	***	13	**	19	**	22	***	20	***	23	***
Niacin	−4	ns	−3	ns	−7	ns	1	ns	3	ns	−3	ns
Vitamin B6	4	ns	4	ns	1	ns	10	*	11	*	4	ns
Folic acid	15	**	13	**	16	*	21	***	19	***	20	*
Vitamin B12	10	*	−4	ns	42	**	18	*	3	ns	52	**
Vitamin C	17	*	13	ns	14	ns	23	*	20	ns	18	***
Vitamin D	34	ns	25	ns	52	ns	39	*	31	ns	54	ns
Vitamin E	7	ns	10	ns	4	ns	13	*	15	**	8	ns
Vitamin A	5	ns	−3	ns	27	*	13	ns	3	ns	39	*
Retinol	−24	ns	−40	*	25	ns	−17	ns	−37	*	37	ns
β-carotene	38	**	41	*	19	**	47	**	49	*	32	**

RD = (RS − nRS) × 100%/nRS; RS (“restricting sugar”)—women who declared “*I cut down on sugar*”, nRS (“not restricting sugar”)—women who declared “*I don’t cut down on sugar*”; SFA—saturated fatty acid; MUFA—monounsaturated fatty acid; PUFA—polyunsaturated fatty acid; *t*-Student test significance level after logarithmic data transformation; * *p* < 0.05; ** *p* < 0.01; *** *p* < 0.001; ns—insignificant differences.

**Table 4:** Relative differences (RD, in %) in mean daily dietary intakes between weekdays and weekend days in women “restricting sugar” (RS) and “not restricting sugar” (nRS): crude and adjusted for 2000 kcal/day models.

Nutrient	Crude (“2 Weekend Days” vs. “5 Week Days”)				Adjusted for 2000 kcal (“2 Weekend Days” vs. “5 Week Days”)			
	RS		nRS		RS		nRS	
	RD	*p*	RD	*p*	RD	*p*	RD	*p*
Energy	37	***	34	***	-	-	-	-
Sucrose	44	***	23	**	6	ns	−8	***
Protein	28	***	28	***	−6	**	−4	**
Fat	39	***	35	***	3	ns	1	Ns
SFA	38	***	30	***	1	ns	−3	Ns
MUFA	43	***	41	***	5	ns	6	Ns
PUFA	39	***	42	***	2	ns	6	Ns
Cholesterol	26	*	16	*	−9	ns	−12	***
Carbohydrates	32	***	30	***	−3	ns	−3	**
Fibre	20	***	22	***	−12	**	−9	***
Sodium	25	**	22	***	−9	**	−9	***
Potassium	17	***	24	***	−15	***	−7	***
Calcium	26	**	32	***	−8	**	−3	Ns
Iron	23	***	23	***	−10	***	−8	***
Zinc	25	***	25	***	−9	***	−5	***
Phosphorus	33	***	17	***	−5	ns	−14	***
Magnesium	23	***	24	***	−11	***	−7	***
Copper	20	***	24	***	−13	***	−7	***
Vitamin B_1_	32	***	31	***	−4	ns	−2	Ns
Vitamin B_2_	23	***	17	***	−10	***	−12	***
Niacin	23	**	27	***	−10	*	−5	*
Vitamin B_6_	21	***	25	***	−11	***	−6	*
Folic acid	22	**	19	***	−10	***	−11	***
Vitamin B_12_	49	ns	0	ns	12	ns	−24	*
Vitamin C	18	ns	17	ns	−15	***	−14	***
Vitamin D	34	*	10	ns	−3	ns	−18	***
Vitamin E	31	***	39	***	−4	ns	2	Ns
Vitamin A	34	ns	2	ns	3	*	−24	***
Retinol	98	**	−5	ns	59	ns	−27	***
β-carotene	−6	ns	11	ns	−30	**	−21	***

RD = (2 weekend days − 5 weekdays) × 100/5 weekdays; RS (“restricting sugar”)—women who declared “*I cut down on sugar”*; nRS (“not restricting sugar”)—women who declared “*I don’t cut down on sugar*”; SFA—saturated fatty acid; MUFA–monounsaturated fatty acid; PUFA—polyunsaturated fatty acid; *t*-Student test significance level after logarithmic data transformation; * *p* < 0.05; ** *p* < 0.01; *** *p* < 0.001, ns—insignificant differences.

## Supplementary Materials

The following are available online at http://www.mdpi.com/2072-6643/10/10/1463/s1, Table S1: Mean daily diet nutritional value (with 95% confidence interval) of women groups: “restricting sugar” (RS) and “not restricting sugar” (nRS) during the week, weekdays and the weekend (*n* = 192): crude and adjusted models.
