# Supplementary material for: “Cutting Down on Sugar” by Non-Dieting Young Women: An Impact on Diet Quality on Weekdays and the Weekend
Source: Nutrients. 2018 Oct 9;10(10):1463. doi: 10.3390/nu10101463 (PMC6213198; doi:10.3390/nu10101463)
Supplement: Supplementary file 1 [file nutrients-10-01463-s001.docx]

**Supplementary material**

**Table S1.** Mean daily diet nutritional value (with 95% confidence interval) of women groups: “restricting sugar” (RS) and “not restricting sugar” (nRS) during the week, weekdays and the weekend (*n* = 192): crude and adjusted models.

|  | Crude | | | | | | | | | | Adjusted for 2000 kcal | | | | | | |
| --- | --- | --- | --- | --- | --- | --- | --- | --- | --- | --- | --- | --- | --- | --- | --- | --- | --- |
| Nutrient (unit) | **7 week days** | | **7 week days** | | | **5 weekdays** | | | **2 weekend days** | | **7 week days** | **7 week days** | | **5 weekdays** | | **2 weekend days** | |
|  | **Total** | | **RS** | **nRS** | | **RS** | **nRS** | | **RS** | **nRS** | **Total** | **RS** | **nRS** | **RS** | **nRS** | **RS** | **nRS** |
| Energy (kcal) | 1601  (1555,1646) | | 1570  (1501,1639) | 1621  (1600,1681) | | 1441  (1377,1505) | 1500  (1443,1555) | | 1971  (1868,2074) | 2011  (1929,2093) | 2000 | 2000 | 2000 | 2000 | 2000 | 2000 | 2000 |
| Sucrose (g) | 44.6  (41.6, 47.7) | 38.3  (33.8, 42.8) | | 48.8  (44.9, 52.7) | 34.6  (30.4, 38.8) | | 46.1  (42.7, 57.0) | 49.9  (43.9, 56.6) | | 56.8  (51.5, 62.1) | 55.4  (52.2, 58.6) | 48.1  (43.1, 53.0) | 60.2  (56.2, 64.2) | 47.2  (42.2, 52.1) | 61.4  (57.0, 65.8) | 50.0  (44.0, 56.0) | 56.2  (51.7, 60.8) |
| Protein (g) | 62.3  (60.3, 64.4) | 64.8  (61.6, 67.9) | | 60.7  (58.1, 63.4) | 60.6  (57.5, 63.7) | | 56.9  (54.4, 59.5) | 77.3  (72.8, 81.9) | | 73.0  (69.2, 76.7) | 78.4  (76.6, 80.2) | 83.5  (80.1, 86.9) | 75.0  (73.2, 76.8) | 85.2  (81.5, 88.9) | 75.9  (74.1, 77.8) | 79.7  (75.7, 83.6) | 72.9  (70.5, 75.4) |
| Fat (g) | 56.1  (54.1, 58.2) | 54.2  (51.1, 57.4) | | 57.3  (54.6, 60.1) | 49.8  (46.6, 53.0) | | 53.0  (50.2, 57.0) | 69.3  (64.6, 74.1) | | 71.4  (67.4, 75.5) | 69.9  (68.5, 71.3) | 69.0  (66.4, 71.7) | 70.5  (68.9, 72.1) | 68.8  (65.9, 71.8) | 70.3  (68.6, 72.0) | 70.6  (67.0, 74.2) | 71.1  (68.2, 73.9) |
| SFA (g) | 21.9  (21.0, 22.8) | 21.1  (19.6, 22.6) | | 22.4  (21.3, 23.6) | 19.4  (17.8, 20.9) | | 21.0  (19.8, 22.1) | 26.7  (24.6, 28.7) | | 27.3  (25.6, 28.9) | 27.3  (26.4, 28.1) | 26.8  (25.4, 28.2) | 27.6  (26.8, 28.5) | 26.7  (25.1, 28.3) | 27.9  (27.0, 28.9) | 27.1  (25.4, 28.8) | 27.1  (25.9, 28.4) |
| MUFA (g) | 21.8  (20.9, 22.8) | 20.7  (19.3, 22.1) | | 22.6  (21.3, 23.8) | 18.9  (17.5, 20.3) | | 20.6  (19.8, 22.8) | 27.0  (24.6, 29.9) | | 29.0  (27.2, 30.9) | 27.2  (26.4, 27.9) | 26.4  (25.1, 27.6) | 27.7  (26.8, 28.5) | 26.2  (24.8, 27.6) | 27.2  (26.3, 28.1) | 27.5  (25.6, 29.3) | 28.8  (27.4, 30.1) |
| PUFA (g) | 8.3  (7.9, 8.6) | 8.3  (7.7, 8.8) | | 8.3  (7.8, 8.7) | 7.6  (7.0, 8.2) | | 7.5  (7.6, 7.8) | 10.5  (9.0, 11.0) | | 10.7  (9.9, 11.5) | 10.3  (9.9, 10.7) | 10.6  (9.9, 11.3) | 10.2  (9.7, 10.6) | 10.6  (9.8, 11.3) | 10.1  (9.6, 10.5) | 10.8  (10.0, 11.7) | 10.7  (9.0, 11.4) |
| Cholesterol (mg) | 245  (232, 258) | 244  (220, 268) | | 245  (230, 260) | 228  (204, 252) | | 237  (221, 236) | 289  (252, 326) | | 275  (250, 299) | 308  (293, 323) | 314  (284, 344) | 304  (288, 320) | 322  (287, 357) | 315  (297, 334) | 294  (258, 330) | 278  (254, 303) |
| Carbohydrates (g) | 220  (213, 227) | 216  (205, 228) | | 223  (214, 232) | 200  (189, 210) | | 208  (200, 216) | 265  (248, 282) | | 271  (259, 283) | 276  (272, 279) | 275  (268, 283) | 276  (272, 280) | 277  (269, 285) | 278  (274, 283) | 269  (259, 279) | 270  (265, 275) |
| Fibre (g) | 16.3  (15.4, 17.1) | 18.9  (17.3, 20.5) | | 14.5  (13.6, 15.5) | 17.9  (16.4, 19.4) | | 13.9  (12.9, 14.9) | 21.6  (19.1, 23.6) | | 16.9  (15.8, 18.0) | 20.6  (19.6, 21.7) | 24.4  22.5, 26.4 | 18.1  (17.1, 19.1) | 25.3  (23.2, 27.1) | 18.8  (17.6, 19.9) | 22.3  (20.4, 24.1) | 17.1  (16.0, 18.1) |
| Sodium (mg) | 1825  (1748,1902) | 1795 (1685,1907) | | 1845  (1739,1952) | 1686  (1586,1785) | | 1737  (1642,1834) | 2100  (1917,2283) | | 2118  (1959,2276) | 2288  (2215,2362) | 2315  2190,2440 | 2271  (2180,2362) | 2381  (2245,2516) | 2314  (2218,2410) | 2161  (1991,2331) | 2110  (1985,2234) |
| Potassium (mg) | 2671  (2587,2756) | 2788  (2653,2924) | | 2595  (2488,2702) | 2668  (2538,2798) | | 2467  (2366,2578) | 3111  (2901,3321) | | 3054  (2906,3201) | 3386  (3290,3482) | 3626  3447,3805 | 3229  (3129,3329) | 3797  3591,4002 | 3321  (3198,3443) | 3213  (3020,3406) | 3073  (2947,3198) |
| Calcium (mg) | 588  (557, 620) | 672  (620, 724) | | 533  (496, 571) | 630  (578, 680) | | 493  (458, 529) | 794  (712, 878) | | 653  (596, 709) | 743  (707, 780) | 871  (805, 937) | 660  (625, 696) | 892  819, 964 | 664  (624, 704) | 817  (737, 897) | 645  (601, 689) |
| Iron (mg) | 9.3  (8.9, 9.6) | 9.7  (9.2, 10.3) | | 9.0  (8.6, 9.4) | 9.2  (8.7, 9.7) | | 8.5  (8.1, 8.9) | 11.3  (10.5, 12.2) | | 10.5  (9.9, 11.1) | 11.7  (11.4, 12.0) | 12.5  (11.9, 13.1) | 11.1  (10.8, 11.4) | 12.9  (12.3, 13.5) | 11.4  (11.0, 11.8) | 11.6  (10.9, 12.3) | 10.5  (10.1, 10.9) |
| Zinc (mg) | 8.1  (7.8, 8.4) | 8.5  (8.1, 9.0) | | 7.8  (7.4, 8.1) | 8.0  (7.6, 8.5) | | 7.3  (6.9, 7.7) | 10.0  (9.3, 10.8) | | 9.2  (8.7, 9.7) | 10.2  (9.9, 10.5) | 11.0  (10.5, 11.5) | 9.6  (9.3, 10.0) | 11.3  (10.8, 11.8) | 9.8  (9.5, 10.2) | 10.3  (9.7, 10.9) | 9.3  (8.9, 9.6) |
| Phosphorus (mg) | 1058  (1018,1099) | 1163  1095,1230) | | 990  (944, 1036) | 1034  (974, 1093) | | 1011  ( 960, 1062) | 1376  (1274,1476) | | 1185  (1122,1248) | 1334  (1292,1376) | 1500  (1427,1574) | 1225  (1186,1263) | 1487  (1379,1596) | 1383  (1305,1460) | 1413  (1332,1494) | 1184  (1139,1230) |
| Magnesium (mg) | 258  (248, 268) | 284  (266, 303) | | 241  (230, 252) | 269  (251, 285) | | 228  (217, 239) | 329  (302, 357) | | 283  (267, 298) | 326  (315, 338) | 367  (346, 388) | 300  (288, 311) | 379  (357, 402) | 307  (294, 320) | 337  (314, 360) | 285  (272, 299) |
| Copper (mg) | 1.02  (0.98, 1.06) | 1.10  (1.03, 1.18) | | 0.97  (0.92, 1.01) | 1.05  (0.98, 1.12) | | 0.92  (0.87, 0.96) | 1.26  (1.16, 1.36) | | 1.14  (1.08, 1.20) | 1.29  (1.24, 1.33) | 1.42  (1.34, 1.51) | 1.20  (1.15, 1.24) | 1.48  (1.39, 1.57) | 1.23  (1.18, 1.28) | 1.29  (1.21, 1.37) | 1.14  (1.09, 1.19) |
| Vitamin B_1_ (mg) | 1.03  (1.00, 1.07) | 1.03  (0.98, 1.09) | | 1.03  (0.98, 1.09) | 0.96  (0.91, 1.02) | | 0.97  (0.92, 1.02) | 1.27  (1.17, 1.36) | | 1.27  (1.19, 1.35) | 1.30  (1.26, 1.36) | 1.33  (1.27, 1.39) | 1.28  (1.23, 1.33) | 1.35  (1.28, 1.41) | 1.29  (1.24, 1.35) | 1.30  (1.22, 1.39) | 1.27  (1.20, 1.34) |
| Vitamin B_2_ (mg) | 1.34  (1.28, 1.39) | 1.46  (1.37, 1.54) | | 1.26  (1.19, 1.33) | 1.37  (1.29, 1.44) | | 1.21  (1.14, 1.29) | 1.69 (1.52,1.85) | | 1.42  (1.34, 1.51) | 1.69  (1.63, 1.76) | 1.90  (1.78, 2.01) | 1.56  (1.49, 1.62) | 1.94 (1.83,2.05) | 1.62  (1.54, 1.70) | 1.75  (1.57, 1.93) | 1.42  (1.35, 1.50) |
| Niacin (mg) | 14.5  (13.9, 15.1) | 14.2  (13.3, 15.1) | | 14.7  (14.0, 15.5) | 13.4  (12.5, 14.4) | | 13.9  (13.1, 14.6) | 16.5  (15.6, 17.7) | | 17.6  (16.4, 18.8) | 18.3  (17.7, 19.0) | 18.4  (17.2, 19.7) | 18.3  (17.5, 19.0) | 19.1  (17.5, 20.6) | 18.6  (17.7, 19.5) | 17.1  (15.6, 18.6) | 17.7  (16.6, 18.7) |
| Vitamin B_6_ (mg) | 1.52  (1.46, 1.57) | 1.56  (1.47, 1.64) | | 1.49  (1.42, 1.57) | 1.47  (1.39, 1.56) | | 1.42  (1.34, 1.50) | 1.79  (1.66, 1.91) | | 1.77  (1.67, 1.88) | 1.93  (1.86, 1.99) | 2.04  (1.91, 2.16) | 1.85  (1.78, 1.93) | 2.10  (1.96, 2.24) | 1.90  (1.81, 2.00) | 1.86  (1.72, 2.00) | 1.78  (1.69, 1.87) |
| Folic acid (ug) | 250  (238, 263) | 272  (251, 293) | | 236  (222, 251) | 256  (236, 275) | | 227  (212, 243) | 313  (280, 345) | | 271  (253, 288) | 318  (303, 334) | 356  (325, 386) | 294  (278, 309) | 365  (336, 394) | 306  (287, 326) | 327  (288, 366) | 272  (256, 287) |
| Vitamin B_12_ (ug) | 3.25  (2.86, 3.64) | 3.43  (2.99, 3.88) | | 3.13  (2.55, 3.71) | 3.00  (2.70, 3.31) | | 3.14  (2.47, 3.81) | 4.46  (3.19, 5.73) | | 3.14  (2.50, 3.80) | 4.12  (3.64, 4.60) | 4.54  (3.82, 5.26) | 3.84  (3.21, 4.48) | 4.30  (3.81, 4.79) | 4.18  (3.35, 5.01) | 4.82  (3.08, 6.57) | 3.18  (2.50, 3.86) |
| Vitamin C (mg) | 95  (87, 102) | 104  (92, 115) | | 89  (78, 99) | 97  (85, 109) | | 86  (74, 96) | 115  (97, 132) | | 100  (87, 114) | 121  (111, 131) | 136  (120,152) | 111  (99, 124) | 140  (122, 159) | 117  (102,133) | 119  (102,136) | 101  (88, 114) |
| Vitamin D (μg) | 2.41  (2.18, 2.65) | 2.85  (2.35, 3.36) | | 2.13  (1.93, 2.33) | 2.58  (2.08, 3.08) | | 2.07  (1.84, 2.30) | 3.46  (2.59, 4.34) | | 2.28  (1.90, 2.67) | 3.07  (2.77, 3.38) | 3.70  (3.04, 4.35) | 2.66  (2.40, 2.93) | 3.67  (2.95, 4.39) | 2.81  (2.48, 3.15) | 3.55  (2.63, 4.48) | 2.30  (1.93, 2.67) |
| Vitamin E (mg) | 7.9  (7.4, 8.3) | 8.2  (7.6, 8,8) | | 7.6  (7.1, 8.2) | 7.7  (7.1, 8.4) | | 7.0  (6.5, 7.5) | 10.1  (9.1, 11.1) | | 9.7  (8.9, 10.6) | 9.86  (9.4, 10.3) | 10.6  (9.8, 11.3) | 9.4  (8.9, 9.9) | 10.8  (9.9, 11.7) | 9.4  (8.8, 10.0) | 10.4  (9.4, 11.3) | 9.6  (8.9, 10.3) |
| Vitamin A (μg) | 936  (827, 1046) | 966  (807,1124) | | 918  (767, 1068) | 884  (764, 1004) | | 913  (740, 1086) | 1188  (806, 1568) | | 935  (756, 1117) | 1190  (1051,1329) | 1281  (1035,1528) | 1130  (964, 1295) | 1265  (1096,1434) | 1231  (1012,1450) | 1297  (768, 1826) | 932  (743, 1121) |
| Retinol (μg) | 444  (350, 540) | 375  (284, 467) | | 491  (345, 637) | 296  (267, 324) | | 495  (324, 666) | 587  (271, 903) | | 471  (318, 623) | 560  (444, 676) | 500  (342, 658) | 599  (437, 762) | 414  (375, 452) | 660  (444, 875) | 660  (215, 1105) | 482  (316, 648) |
| β-carotene (μg) | 2948  (2622,3276) | 3540  2853,4226) | | 2562  (2272,2852) | 3524  (2796,4251) | | 2508  (2205,2811) | 3316  (2712,3921) | | 2782  (2192,3370) | 3776  (3365,4187) | 4685  (3807,5563) | 3180  (2845,3515) | 5103  (4081,6124) | 3428  (3003,3852) | 3565  (2841,4288) | 2697  (2182,3212) |

RS (“restricting sugar”)—women who declared “*I cut down on sugar”,* nRS (“not restricting sugar”)—women who declared “*I don’t cut down on sugar”*; SFA—saturated fatty acid, MUFA—monounsaturated fatty acid, PUFA—polyunsaturated fatty acid.
